# The impact of train-the-trainer programs on the continued professional development of nurses: a systematic review

**DOI:** 10.1186/s12909-023-04998-4

**Published:** 2024-01-04

**Authors:** Mette Andersen Nexø, Natassia Rosewood Kingod, Signe Hornsleth Eshøj, Emilie Mølholm Kjærulff, Ole Nørgaard, Tue Helms Andersen

**Affiliations:** 1 Department of Education, Copenhagen University Hospital – Steno Diabetes Center Copenhagen, Borgmester Ib Juuls Vej 83, Herlev, Denmark; 2 Department of Public Health, Faculty of Health and Medical Sciences, University of Copenhagen, Øster Farimagsgade 5, Building 24 Q, 1 Floor, Copenhagen K, Denmark

**Keywords:** Systematic review, Train-the-trainer, Nurses, Knowledge, Learning outcomes

## Abstract

**Background:**

Train-the-trainer (TTT) programs are widely applied to disseminate knowledge within healthcare systems, but evidence of the effectiveness of this educational model remains unclear. We systematically reviewed studies evaluating the impact of train-the-trainer models on the learning outcomes of nurses.

**Methods:**

The reporting of our systematic review followed PRISMA 2020 checklist. Records identified from MEDLINE, Embase, CINAHL, and ERIC were independently screened by two researchers and deemed eligible if studies evaluated learning outcomes of a train-the-trainer intervention for trainers or trainees targeting nurses. Study quality was assessed with Joanna Briggs Institute’s critical appraisal tools and data of study characteristics extracted (objective, design, population, outcomes, results). Heterogeneity of outcomes ruled out meta-analysis; a narrative synthesis and vote counting based on direction of effects (*p* < 0.05) synthesized the results.

All records were uploaded and organized in EPPI-Reviewer.

**Results:**

Of the 3800 identified records 11 studies were included. The included studies were published between 1998 and 2021 and mostly performed in the US or Northern Europe. Nine studies had quasi-experimental designs and two were randomized controlled trials. All evaluated effects on nurses of which two also included nurses’ assistants. The direction of effects of the 13 outcomes (knowledge, *n* = 10; skills, *n* = 2; practice, *n* = 1) measured in the 11 included studies were all beneficial. The statistical analysis of the vote counting showed that train-the-trainer programs could significantly (*p* < 0.05) improve trainees’ knowledge, but the number of outcomes measuring impact on skills or practice was insufficient for synthesis.

**Conclusions:**

Train-the-trainer models can successfully disseminate knowledge to nurses within healthcare systems. Considering the nurse shortages faced by most Western healthcare systems, train-the-trainer models can be a timesaving and sustainable way of delivering education. However, new comparative studies that evaluate practice outcomes are needed to conclude whether TTT programs are more effective, affordable and timesaving alternatives to other training programs.

**Trial registration:**

The protocol was registered in Research Registry (https://www.researchregistry.com, unique identifying number 941, 29 June 2020).

**Supplementary Information:**

The online version contains supplementary material available at 10.1186/s12909-023-04998-4.

## Background

Train-the-trainer (TTT) programs were originally used by non-governmental organisations and universities in the 1970s as an educational model delivering cost-effective education to hard-to-reach populations in settings with limited resources [1, 2]. Drawing on the assumptions that social capital from relationships within a community optimize the learning process [3], local trainers familiar with the local language, culture, and economic realities were employed to educate their peers [4, 5]. TTT models have subsequently been applied across disciplinary fields and within various healthcare contexts and clinical settings [5–9] to update healthcare professionals’ knowledge and skills and implement evidence-based medical practices [10, 11].

**Fig. 1:**
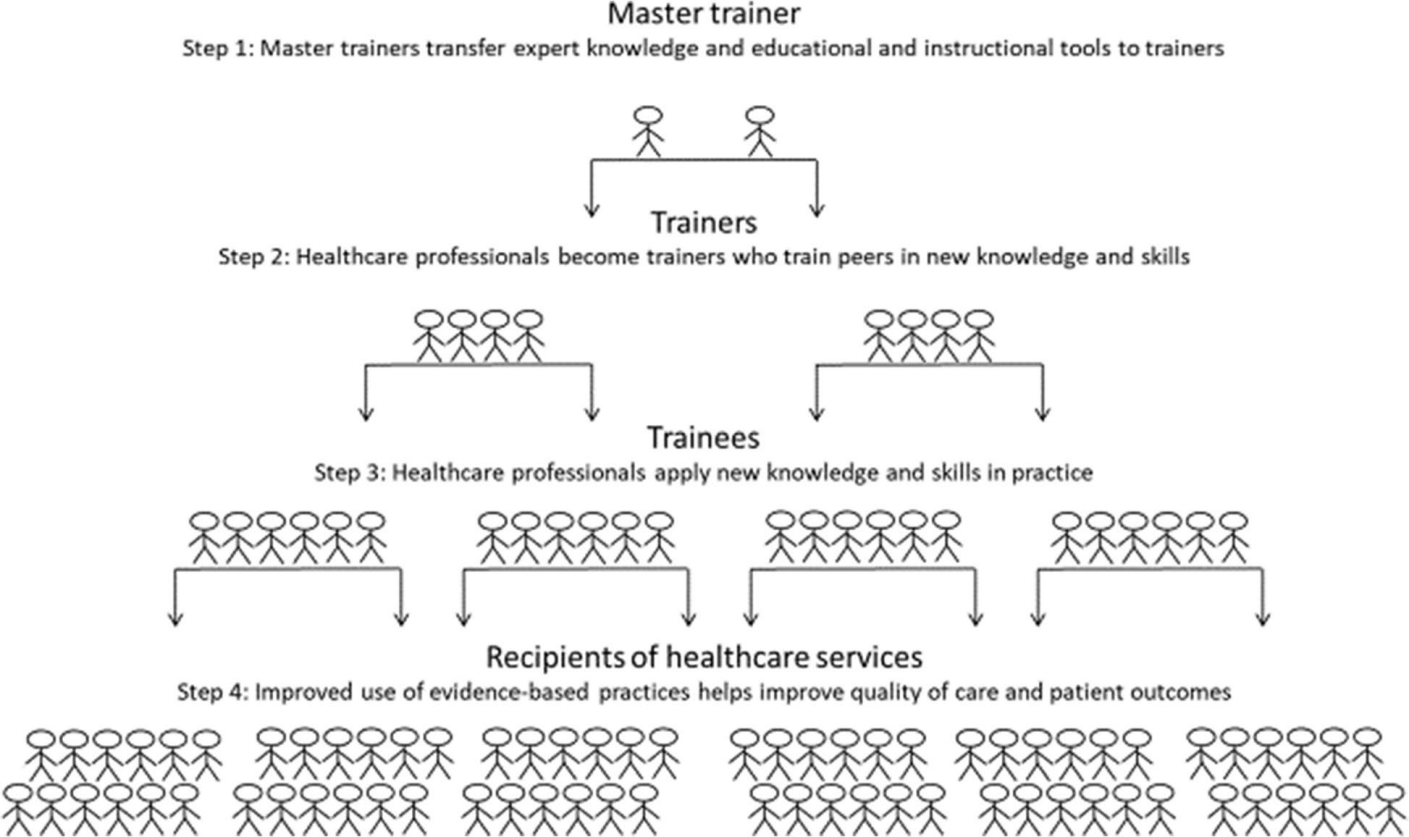
Key elements of train-the-trainer models in healthcare contexts

Although TTT programs can draw on a wide range of educational and implementation strategies, several steps characterize knowledge dissemination in healthcare contexts (Fig. 1).

Master trainers with appropriate expertise educate selected professionals, preparing them to train others. Traditionally, trainers are often nurses or social workers working in the organization where the TTT program is implemented. Trainers learn about new expert knowledge, instructional tools, and guidelines, which they then disseminate to ‘trainees’, i.e., their professional peers [1]. No single gatekeeper of knowledge exists because expert knowledge, skills and evidence-based practices are disseminated across many professionals [1]. Ultimately, the application of trainees’ newly acquired skills can help ensure quality of care and better treatment for recipients of healthcare services. Advantages of using professional peer trainers include availability of support during the workday and insight into organization characteristics that can help trainees overcome barriers to applying new knowledge and skills in practice [3].

Since TTT model elements have been linked to improved clinical teamwork [12] and higher job satisfaction and decreased staff turnover [13], the TTT model may be a more efficient alternative to traditional direct trainer models in which more experienced professionals provide informal training of specific skills. Also, given their potential to deliver continual peer to peer support throughout the workday, TTT models may prove more sustainable and cost-effective than other training models [14].

An increasing number of studies investigating the impact of TTT models in healthcare settings have been published in the past decade and three systematic reviews have synthesized findings across studies [2, 15, 16]. Anderson and Taira [2] found evidence showing that TTT models could propagate knowledge and skills for providers in limited resource settings, but further research was needed to infer whether the model was sustainable for the long term. Two other reviews focused on health and social care workers. In a narrative synthesis, Pierce et al. [15] found that TTT programs applying a blended learning approach that combined interactive and multifaceted methods were the most effective to disseminate knowledge to healthcare and social care professionals. A meta-analysis concluded that TTT programs improved trainers’ health and social care knowledge domains [16]. However, this review did not focus on the impact on trainees’ knowledge, which is an essential feature that distinguishes the TTT programs from other training models. Although these systematic reviews provide insights into the effectiveness of TTT programs, to our knowledge, no systematic review has considered the claimed potential of TTT programs to disseminate knowledge, like a waterfall, from expert to trainee, through the different steps that require different training elements and qualities of teachers (Fig. 1).

Today, most Western healthcare systems face staff shortages and high work pressure [17–19]. The qualities inherent of TTT models can be an effective and sustainable way of disseminating knowledge. However, a new updated review is needed to clarify the evidence for these qualities and, in so doing, help healthcare providers make evidence-based decisions regarding the best way of delivering and implementing education in high strung healthcare systems. The aim of this systematic review was to synthesize findings about the impact of TTT models, disseminating knowledge from trainers to trainees, on nurses’ learning outcomes.

## Methods

The reporting of our systematic review followed the Preferred Reporting Items for Systematic Reviews and Meta-Analyses (PRISMA) 2020 checklist, and the reporting of the literature search followed the extension PRISMA Statement for Reporting Literature Searches in Systematic Reviews (PRISMA-S) [20] The protocol was registered in Research Registry, 29 June 2020 (https://www.researchregistry.com, unique identifying number 941). The reporting of the analysis followed Synthesis Without Meta-analysis (SWiM) reporting guidelines [21].

### Eligibility criteria

The research question, eligibility criteria and search strings were structured using the Population, Intervention, Comparison, Outcome (PICO) framework. Records were eligible for inclusion if they: 1) targeted nurses, social and healthcare assistants, or healthcare assistants alone (P), 2) described a TTT intervention or program and specified how knowledge was transferred from master trainer to trainer or from trainer to trainee (I), and 3) evaluated intervention learning outcomes (i.e., attitudes, knowledge, skills and practice) for nurses in a healthcare context (O). A preliminary search identified no randomized controlled trials (RCTs), and controlled trials and pre-/post-intervention studies were thus also included. Consequently, no comparison (C) was necessary, but could be other educational models. For inclusion in the synthesis, records had to represent primary studies published in peer-reviewed journals.

### Search strategy

We conducted a literature search for studies published from inception to 21 January 2020 in MEDLINE (Ovid), Embase (Ovid), CINAHL (EBSCO) and ERIC (EBSCO). The search was updated in all four databases on 10 September 2021. The literature was searched for the two key concepts of ‘train-the-trainer’ and ‘health personnel’ using controlled vocabularies (e.g., medical subject headings), free-text terms, and keywords when possible (i.e. title, abstract, keywords and MeSH terms).

We applied an RCT filter that was adapted to include a broader range of studies evaluating impact. A filter removing animal studies was used in MEDLINE and Embase. Due to the relatively low number of studies retrieved in CINAHL and ERIC, we chose not to apply filters in those databases. A limit excluding MEDLINE journals was applied in Embase to avoid duplicate journals. Two information specialists (THA and ON) developed and conducted the literature search. The search strategy was evaluated by testing its ability to identify known key articles. The complete search strategy is available in Additional file 1.

All records were uploaded to and organized in EPPI-Reviewer [22]. Deduplication was carried out in EPPI-Reviewer using the built-in automated deduplication function supplemented by a manual search for duplicate records.

### Study selection

All records were screened in duplicate and independently by title and abstract in EPPI-Reviewer, each by two authors (NK, SHE, EMK). Included full-text reports were assessed for eligibility by two authors (NK, SHE). Disagreements were resolved by discussion and in two cases resolved by a third author (THA).

Full-text reports were retrieved electronically. If no electronic version was available or could not be retrieved via a research library (Royal Danish Library), we e-mailed the corresponding author. If no response was received within a month, the report was excluded. References of included studies were searched to identify any additional relevant references. Records in languages other than English, Danish, Swedish and Norwegian (languages understood by the review team) that we considered relevant based on title and abstract were not included in the synthesis but have been listed in Additional file 2 for others to analyze.

### Data extraction

Two authors (SHE and NK) designed a data extraction form which was pilot tested and adapted accordingly before final data extraction. One author (SHE) extracted data from included reports which was checked by a second author (NK). In the case of missing data, an e-mail inquiry was sent to the first or corresponding author. If missing data were essential to include in the synthesis and no response was received after 1 month, the report was excluded.

### Quality assessment

Reports included after the full-text screening were assessed for methodological quality by two authors (SHE, NK) using the Joanna Briggs Institute (JBI) critical appraisal tools (CAT) for quasi-experimental studies and RCTs [11]. A pilot search revealed few RCTs and many quasi-experimental studies with a wide range of study designs. To allow for a robust synthesis, quasi-experimental studies that failed to meet criteria for comparison due to insufficient reporting of data were excluded before analysis. More specifically, studies that were rated ‘no’ or ‘unclear’ to JBI quality assessment check list item 7 (similar outcome measurements for compared groups), 8 (reliable outcome measures), and 9 (appropriate statistical analysis) were excluded (Table 2). To help overview the quality assessment scores we calculated a percentage score for each study by dividing the number of CAT items with ‘yes’ responses by the total number of items [23].

### Data synthesis

To distinguish between the impact of TTT interventions on learning for trainers and trainees, we grouped studies by their target population groups. Any impact on trainers’ learning was regarded the result of training by master trainers. Any impact on nurses’ (trainees) learning was attributed the effect of training by trainers. We therefore also regarded a successful training of trainees a result of a successful training of trainers by the master trainers (Fig. 1).

Characteristics of included studies ruled out conducting a meta-analysis. Only four of eleven included studies reported measures of precision. Effect measures varied across studies and nine studies did not report *P* values, precluding summarizing effect estimates or combining *P* values. In addition, outcome definitions differed substantially across studies. As an alternative, we synthesized findings by vote counting based on direction of effect, as specified in the Cochrane Handbook for Systematic Reviews of Interventions [24]. Regardless of statistical significance, the direction of the effect of TTT intervention on each independent study outcome was counted as beneficial if data indicated a positive effect (‘1’) or as not beneficial if data indicated no effect or a negative effect on the outcome (‘0’). Effect direction was based on pre- and post-intervention measures, not the results of comparison with any control group. To examine the statistical significance of effects by vote count and help clarify the certainty of the findings, we conducted binomial tests (one sample, non-parametric test) comparing the number of beneficial and not beneficial direction of effects for individual outcomes (e.g., knowledge) and all outcomes (knowledge, skills, and practice) on trainers, trainees, and trainers and trainees together. Statistical significance was set at *P* < 0.05 (two-tailed) and 95% confidence intervals with Clopper-Pearson interval. Data was analyzed by SPSS Statistics for Windows version 25.

## Results

The literature search yielded 3800 records. Duplicates automatically marked by EPPI-Reviewer (*n* = 229) were manually verified; manual screening detected an additional 31 duplicate records. Of all remaining records (*n* = 3540) screened by title and abstract, 3332 were excluded. Full-text reports for two of the resulting 208 records could not be retrieved through correspondence with the authors. Four studies were in other languages than English, Danish, Swedish and Norwegian, 12 studies were not journal articles, and two full-text records were unavailable.

**Fig. 2:**
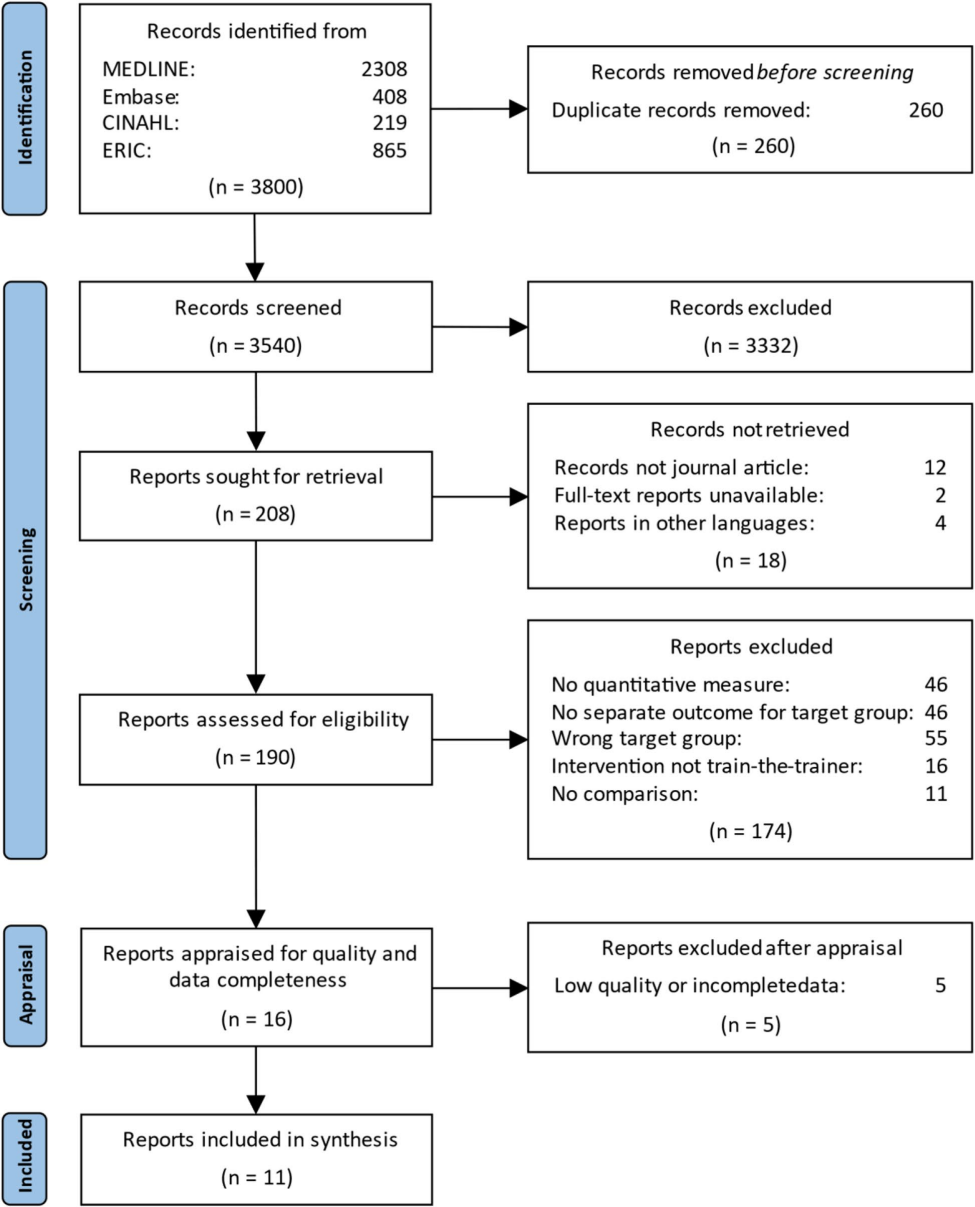
Flow diagram of study selection

Of 190 records assessed for eligibility, 16 met inclusion criteria. After critical appraisal, five were excluded [25–29]. Eleven studies were included for data extraction and synthesis. Figure 2 provides additional details about the screening and selection process.

### Study characteristics

#### Demographics

The 11 included studies were published between 1998 and 2021 (Table 1). Four studies were conducted in the US [13, 30–32], three in Northern Europe [33–35], one in South Asia [6], and one in the Middle East [36]. Two studies did not report the country where the TTT intervention took place [37, 38]. Nine studies were quasi-experimental [6, 13, 30–32, 34, 36–38] of which two included a control group [31, 34].

**Table 1:** Key characteristics of included studies

**Author (year), country**	**Objective/aim**	**Study design**	**Study population and setting**	**Outcomes**	**Results**	**Direction of effect**^a^
Trainee (Transfer of knowledge, skills or practice from trainer to trainee)						
Anderson et al. [37] *USA*	To implement and evaluate a palliative care professional development program for ICU bedside nurses.	Quasi-experi-mental	428 nurses 10 ICUs and 5 health centers	Skills: Percentage self-reporting ‘very good’ or ‘excellent’ skills on 15 tasks pre- and post- training.	Improved skills^b^: Mean percentage change: 35.07, *P* < 0.01	Beneficial (skills)
de Beuers et al. [33] *Netherlands*	Examine the effect of a TTT intervention and implementation as usual versus only implementation as usual	Cluster RTC	*Intervention group:* 121 mental health nurses *Control group:* 51 mental health nurses Psychiatric departments	Knowledge: Mean score on trainees’ self-evaluation of patients’ suicidal behavior (7 items, total score range, 7–35) Practice: Mean percent correct on 125 items on guideline adherence; correct responses to 5 videoclips of interactions between healthcare professionals and suicidal characters (pre- and 3 months post-training, intervention vs. control).	Improved knowledge: Mean difference 2.7 (95% CI: 1.7–3.8, *P* < 0.001) Improved practice: Mean difference 6.6 (95% CI: 3.2–10.0, *P* < 0.001) (both outcomes adjusted for department and baseline score)	Beneficial (knowledge) Beneficial (practice)
Kalisch et al. [13] *USA*	To test the impact of a TTT intervention on the level of satisfaction with nursing teamwork and the amount of missed nursing care.	Quasi-experi-mental	242 nurses Inpatient hospital units	Knowledge: Mean number of correct answers (range, 0–15) to knowledge test pre, post and 2 months after training.	Improved knowledge: Mean difference pre/post: 0.51 (95% CI: 0.19; 0.84) 2 months: 0.40 (95% CI: 0.06; 0.74) *P* = 0.005	Beneficial (knowledge)
Nyamathi et al. [6] *India*	To evaluate the effectiveness of a TTT program in enhancing knowledge of prevention, transmission, and treatment of HIV and counseling among nurses in a major government-sponsored medical hospital in Delhi, India.	Quasi-experi-mental	100 nurses Tertiary-care public hospital	Knowledge: Mean score (range, 0–21) on 21 items testing knowledge pre- and post-training.	Improved knowledge: Pre-training: 12.8 (SD, 4) Post-training: 16.4 (SD, 4.15), *P* < 0.001	Beneficial (knowledge)
Ramberg and Wasserman [34] *Sweden*	To assess whether implementation of suicide preventive activities in psychiatric healthcare clinics by key persons had any effect on the staff’s perception of being sufficiently trained for their work with patients who had attempted suicide, and their perception of clarity in this work.	Quasi-experimental with control group	*Intervention group:* 103 nurses and nursing assistants *Control group:* 135 nurses and nursing assistants 11 psychiatric healthcare clinics	Knowledge: Percent of self-ratings of being ‘sufficiently trained’ and perceiving their ‘work made difficult by lacking knowledge’ pre and 1.5 years into the 2-year program.	Improved knowledge ‘Sufficiently trained’ self-ratings among nurses and nursing assistants, pre/post: Intervention group: 52.3/54.5, *P* = 1.00; 23.7/47.5, *P* = 0.003 Control group: 45.8/43.8, *P* = 1.00, 37.2/31.0, *P* = 0.458 Decreased ‘lack of knowledge’ among nursing assistants, pre/post: Intervention group: 52.5/30.5, *P* = 0.01 Control group: 54.0/50.6, NS Pre/post between-group difference,* P* < 0.016	Beneficial (knowledge)
Rholdon et al. [32] *USA*	To evaluate the effectiveness of the implementation of a simulation-learning based training using a TTT model on the acquisition and retention of knowledge about infant safe sleep practices among nursing staff employed at a women’s and children’s hospital located in southwest Louisiana.	Quasi-experi-mental	74 nurses Specialty women’s and children’s hospital	Knowledge: Mean score on 11-question pre/post knowledge test (KR-20 = 0.60)	Improved knowledge Pre-training: 0.6 Post-training: 0.78 3 months after training: 0.81 *P* < 0.001	Beneficial (knowledge)
Warming et al. [35] *Denmark*	To evaluate the effect of a transfer technique education program alone (TTT) or in combination with physical fitness training compared with a control group following their usual routine.	Cluster RTC	*Intervention group:* 86 nurses *Control group:* 51 nurses 11 wards at a university hospital	Knowledge: Mean score (range, n/a) on 4 items about what to do in four situations pre-training and 12 months later.	Increased knowledge, pre/post: Intention-to-treat analysis Interrvention group: 11.45/13.06, NS Control group:10.8/11.91, NS *P* values not reported Per protocol analysis Intervention group: 11.10/13.33 Control group: 10.78/ 11.90 at 12 months, *P* = 0.045	Beneficial (knowledge)
Trainers (transfer of knowledge or skills from master trainer to trainer)						
Smith et al. [31] *USA*	Explore the generalizability of a geriatric mental health training program that was used successfully with a culturally homogeneous group of long-term care providers in the rural Midwest USA.	Quasi-experimental with control group	*Trainers:* 31 nurses *Trainees:* 92 nurses and nurse aides 14 long-term care facilities *Control (direct trainer):* 101 nurses and nurse aides 10 long term care facilities	Knowledge: Mean score on 77-item pre/post knowledge test	Increased knowledge, pre/post Intention-to-treat analysis Intervention group: 50.67/51.5, *P* = 0.51 Control group: 52.6/56.4, *P* < 0.005 Direct trainer superior to TTT, *P* < 0.0001	Beneficial (knowledge)
Carta et al. [38] *Country not reported*	To report on: • a health education program on patient handling techniques carried out among nurses and healthcare assistants • a TTT program to implement safer patient handling techniques within the framework of a multidisciplinary intervention.	Quasi-experi-mental	48 nurses Hospital	Knowledge: Mean score (range unclear) on 20-item test pre- and 1 month post-training Skills: Mean score (range, 0–30; three skills scored 0–10) based on observation pre- and 1 month post-training).	Improved knowledge, pre/post: 29.8/33.5, *P* < 0.05 Improved skills, pre/post: Supine transfer board: 26.5/29.9, *P* < 0.05 Transfer holding belts: 11.1/27.4, NS Hoist: 25.9/28.8, NS	Beneficial (knowledge) Beneficial (skills)
Karayurt et al. [36] *Turkey*	To describe curriculum, development and evaluation of a breast cancer TTT program.	Quasi-experi-mental	82 nurses Hospitals and community health centers	Knowledge: Mean score (range, 0–100) on 25-item knowledge test pre- and post-training.	Improved knowledge, pre/post: 55.7/80.1, *P* < 0.001	Beneficial (knowledge)
Sinvani et al. [30] *USA*	To describe and evaluate an innovative multicomponent delirium CAM-ICU champion education and training. program to improve delirium detection by critical care nurses across a large integrated health system.	Quasi-experi-mental	63 nurses 14 ICUs	Knowledge: Percent of correct completions on five delirium cases on 6-item questionnaire pre- and post-training.	Mean percent of correct completions improved 12.95% on 9 of 30 items (*P* < 0.05)	Beneficial (knowledge)

*Abbreviations*: *CAM* Confusion assessment method, *CI* Confidence interval, *HIV* Human immunodeficiency virus, *ICU* Intensive care unit, *NS* Not statistically significant, *SD* Standard deviation

^a^Regardless of statistical significance levels, the effect direction for each independent outcome was counted as beneficial if data indicated an improvement or not beneficial if data indicated a negative effect. This was also the case if TTT intervention was not superior in comparison with the control group

^b^Anderson et al. [37] and Sinvani et al. [30] reported outcomes as results of each individual questionnaire item and multiple features of each case presented with no confidence intervals. For simplicity, we calculated the mean percentage change for items and cases for these studies. For Anderson et al. [37], we calculated the mean percentage change for each item. Sinvani et al. [30] reported a mean increase for each feature in each case, and the total mean was calculated directly from these

Two studies were cluster RCTs [33, 35]. One quasi-experimental study compared the TTT intervention to a direct trainer teaching intervention [31] and the remaining studies had no comparison intervention [33–35].

All studies investigated the effect on nurses [6, 13, 30–38] and two studies also included nursing assistants or aides [31, 34].

The collective study population of included studies was 1808 (range of individual study populations: 8–428). Eight studies were conducted in hospital settings [6, 13, 30, 32, 35–38]; two of these also included health centers [36, 37]. Two studies took place in psychiatric settings [33, 34] and one was conducted in long-term care facilities [31].

The specific knowledge or skills of the TTT programs varied, but most studies examined psychiatric knowledge or skills [30, 31, 33, 34] or prevention of low back pain [35, 38]. Among other topics included palliative care [37], HIV counselling [6], and infant safe sleep practices [32].

#### Outcomes

Knowledge was the most common outcome measure, used in ten studies [6, 13, 30–36, 38]. One study assessed clinical practice [33] and two studies measured skills [37, 38]. No studies investigated the effect of the TTT intervention on attitudes. In included quasi-experimental studies, outcomes were most commonly measured by items testing attendees’ knowledge pre- and post-intervention.

Six studies defined knowledge as the score on knowledge tests [6, 13, 31, 32, 36, 38]. In one of these studies, the score was defined as the number of correct answers [13], whereas three other studies calculated knowledge scores in various ways [32, 36, 38]. In the remaining studies defining knowledge as a test score, it was unclear if the score was calculated or reflected the number of correct answers [6, 31].

The remaining four studies measured knowledge through self-evaluation [33], perceptions of being sufficiently trained or lacking knowledge [34], correct responses about what to do in various situations [35], and correct completion of cases [30]. Practice was defined as guideline adherence and measured by correct responses to videoclips [33], whereas skills were measured as self-evaluation of skills on different tasks [37] or as a score based on observation [38].

#### Impact on outcomes

Seven studies measured the effect of the TTT programs on trainees [6, 13, 32–35, 37] and four studies measured the effect on trainers [30, 31, 36, 38].

Six reports included mean pre- and post-intervention scores for the intervention group [6, 31, 32, 35, 36, 38]. Two of these also included mean pre- and post-intervention scores for a control group [31, 35]. Another study reported the mean pre- and post-intervention difference in scores for the intervention group [13]. Only one study calculated the mean difference-in-difference [33]. The remaining three studies measured outcomes as percentages. Two reported only pre- and post-intervention percentages of the desired outcome for either the intervention group [37] or both the intervention and control groups [34]. The third study reported a percentage increase from pre- to post-intervention for the intervention group [30].

Six studies measured pre- and immediate post-intervention outcomes [6, 30, 31, 34, 36, 37]. One of these studies measured post-intervention outcomes 1.5 years into a 2-year program [34]. Three studies measured outcomes before and 1, 3 or 12 months after the intervention, rather than immediately post-intervention [33, 35, 38]. The remaining two studies measured pre-, post- and follow-up outcomes at 2 and 3 months after the TTT intervention [13, 32].

### Quality assessment

Quality appraisal of all 16 studies is shown in Table 2 (quasi-experimental studies) and Table 3 (RCTs). In accordance with the pre-defined quality assessment criteria, we excluded five quasi-experimental studies due to poor quality [25–29] (Table 2) and included all the studies with design RCT [31, 35] (Table 3). Consequently, of the 16 studies, 11 were included in the final synthesis. The nine quasi-experimental studies included in the synthesis were of good quality, with 67–89% positive responses to quality appraisal items. Studies with the lowest scores did not apply a control group, use multiple measures, or present complete follow-up data or results (Table 2).

**Table 2:** Quality assessment of quasi-experimental studies

	Appraisal results for individual JBI checklist items									Total quality score (%)^b^
	1	2	3	4	5	6	7	8	9	
	Clarity about cause and effect	Similar participants in comparisons	Similar treatment other than intervention	Control group	Multiple outcome measurements	Follow up complete or described and analyzed	Similar outcome measurements for compared groups	Reliable outcome measures	Appropriate statistical analysis	
Included studies after the quality assessment										
Anderson et al. (2017) [37]	Y	Y	Y	N	N	Y	Y	Y	Y	78
Carta et al. (2010) [38]	Y	Y	Y	N	N	Y	Y	Y	Y	78
Kalisch et al. (2013) [13]	Y	Y	Y	N	Y	Y	Y	Y	Y	89
Karayurt et al. (2010) [36]	Y	Y	Y	N	N	Y	Y	Y	Y	78
Nyamathi et al. (2008) [6]	Y	Y	Y	N	N	Y	Y	Y	Y	78
Ramberg and Wasserman (2004) [34]	Y	Y	Y	Y	N	Y	Y	Y	Y	89
Rholdon et al. (2020) [32]	Y	Y	Y	N	Y	N	Y	Y	Y	78
Sinvani et al. (2021) [30]	Y	Y	Y	N	N	N	Y	Y	Y	67
Smith et al. (1998) [31]	Y	Y/N^c^	Y/Y^c^	Y	N	N	Y/Y^c^	Y	Y	72
Excluded studies after the quality assessment										
Goucke et al. (2015) [25]	Y	Y	Y	N	N	Y	Y	Y	N	Excluded^a^
Hanssens et al. (2006) [26]	Y	U	Y	N	N	N	U	U	N	Excluded^a^
Hardy and Kingsnorth (2015) [27]	Y	Y	Y	N	N	U	Y	Y	U	Excluded^a^
Jacobs-Wingo et al. (2019) [28]	Y	Y	Y	N	N	U	Y	Y	U	Excluded^a^
Lombardo et al. (2002) [29]	Y	Y	Y	N	N	N	Y	Y	N	Excluded^a^

*Abbreviations*: *JBI* Joanna Briggs Institute, *N* No, *U* Unclear, *Y* Yes

^a^Studies with ‘no’ or ‘unclear’ appraisals for questions 7–9 were excluded

^b^Number of ‘yes’ appraisals divided by the total number of available appraisals (*n* = 9). Y/Y appraisals counted as a single ‘yes’, Y/N counted as 0.5 of a single ‘yes’

^c^Smith et al. [31] studied the effect pre- and post-intervention and compared with a direct trainer intervention (control). The appraisals are recorded here as pre and post/intervention and control group comparisons

**Table 3:** Quality assessment of randomized controlled trials with Joanna Briggs Institute’s critical appraisal tools

	Appraisal results for individual JBI checklist items													Total quality score (%)^a^
	1	2	3	4	5	6	7	8	9	10	11	12	13	
	True randomization	Concealment of allocation	Treatment groups similar at baseline	Blinding of participants	Blinding of those delivering treatment	Blinding of outcome assessors	Identical treatment other than intervention of interest	Follow up complete or described and analyzed	Intention-to-treat analysis performed	Same outcome measurements for treatment groups	Reliable outcome measures	Appropriate statistical analysis	Appropriate trial design	
De Beurs et al. (2015) [33]	U	U	N	n/a	n/a	N	Y	Y	Y	Y	Y	Y	Y	64
Warming et al. (2009) [35]	U	U	N	n/a	n/a	N	Y	Y	Y	Y	Y	Y	Y	64

*Abbreviations*: *JBI* Joanna Briggs Institute, *N* No, *n/a* Not available, *U* Unclear, *Y* Yes

^a^Number of ‘yes’ appraisals divided by the total number of available appraisals (*n* = 11)

The two RCT studies were of lower overall quality than the quasi-experimental studies (Table 3). It was unclear whether randomization could be regarded as ‘true’ and whether treatment allocation was concealed (Table 3). Two questions regarding blinding of participants or those delivering treatment were not scored because they were not applicable to a TTT intervention and did not count in final percentage scores. In addition to JBI appraisal criteria, it is worth noting that only one quasi-experimental study and one RCT [33, 38] adjusted estimates for important confounders (e.g., department, baseline score, sex, age education, length of employment, and job title) and both RCTs included in the synthesis conducted intention-to-treat analyses [31, 35].

### Synthesis

The 11 included studies collectively reported 13 effect directions (standardized metrics), all of which were beneficial, indicating that the TTT model can increase knowledge, skills and practice in all target groups we identified (Table 4).

**Table 4:** Binomial tests of vote counts

	Effect direction		Estimate	95% confidence interval	*P* value^a^
	Beneficial	Not beneficial			
Knowledge^b^					
Trainees	6	0	1.00	0.54–1.00	0.031
Trainers	4	0	1.00	0.40–1.00	0.125
Trainers and trainees	10	0	1.00	0.69–1.00	0.002
Knowledge, skills, and practice					
Trainees^c^	8	0	1.00	0.63–1.00	0.008
Trainers^d^	5	0	1.00	0.40–1.00	0.125
Trainers and trainees^e^	13	0	1.00	0.75–1.00	0.001

^a^Two-tailed

^b^Knowledge was the only individual outcome with sufficient cases for testing

^c^Knowledge, 6 outcomes; practice, 1 outcome; skills, 1 outcome

^d^Knowledge, 4 outcomes; skills, 1 outcome

^e^Knowledge, 10 outcomes; skills, 2 outcomes; practice, 1 outcome

Trainers significantly improved knowledge for trainees (6 metrics, *P* < 0.031) or both trainees and trainers (10 metrics, *P* < 0.002). Although all four included metrics were beneficial, knowledge was not significantly transferred from master trainers to trainers (*P* = 0.125). Too few metrics were reported for the outcomes of skills (two metrics) and practice (one metric) to conduct binomial testing. However, pooling skills and practice with knowledge yielded similar results. TTT models significantly improved outcomes for trainees (*P* < 0.008) and both trainers and trainees (*P* < 0.001) but not trainers (*P* < 0.125).

## Discussion

All effect directions of included studies suggested that TTT interventions can improve knowledge, skills, or practice. However, only for knowledge transfer between trainer and trainee was the number of effect directions sufficient to detect a statistically significant increase. Too few effect directions to permit testing for statistical significance were reported for knowledge transfer from master trainer to trainer and the impact of TTT interventions on skills and practice.

Although our findings are consistent with those of one previous systematic review [15], Pearce et al. [15], or the two other previous reviews [2, 16] did not distinguish between the different levels of the TTT program (e.g., knowledge dissemination from master trainer to trainer, from trainer to trainee). Also, our synthesis was based on an updated literature search and the vote count methodology enabled us to account for the summarised direction of effects.

Most learning outcomes of TTT programs can be evaluated in light of Kirkpatrick’s framework [39], distinguishing between their impact on trainees’ reactions (e.g., feelings about the program), learning (e.g., knowledge, skills, attitudes), behavior (e.g., performance in practice), and results (e.g., organizational benefits or patient outcomes). Most studies included in our synthesis evaluated learning outcomes, primarily by knowledge. Presumably because knowledge outcomes are easy to measure with self-reported questionnaires and requires a limited follow up period. Only a single study assessed behavioral outcomes (performance in practice) and none evaluated results. Changes in knowledge may not lead to changed behavior or better care [40]. Study designs that include long-term follow-up measurements could provide insights into whether TTT interventions lead to behavior change or improve care, generating more robust findings about their effects on practice.

None of the included studies investigated how the program was taught and generally lacked transparency about assumptions of how knowledge was best transferred. Improved methodological, theoretical and pedagogical frameworks in future evaluation frameworks are warranted to further illuminate the effectiveness of TTT programs on different aspects of practice and care. For example, study designs that include theoretical frameworks (e.g., Kirkpatrick’s learning model) can help explain how and why TTT-programs impact outcomes at different levels of learning (e.g., reaction, learning, behavior or results).

Our synthesis distinguished between the impact of TTT interventions on trainers and trainees. It can be argued that the impact of learning of TTT trainers is the same as in a direct trainer intervention. However, trainers of TTT programs are both subjects and agents of change, which introduces complexity that distinguishes TTT models from direct training models [41]. Also, trainers are often selected to be trainers because they already have a high level of experience or knowledge and therefore would be expected to improve less on tests of knowledge. One study included in our review found that direct training was superior to the TTT model on trainees’ knowledge test scores [31], suggesting that other ways of transferring knowledge to trainees may be more effective [15].

### Strengths and limitations

To the best of our knowledge, our review is the first to distinguish between outcomes for trainers and trainees in synthesising the findings which can help decision makers evaluate the benefits of TTT models (e.g., cost-effectiveness or peer-facilitation) in light of limited evidence of their effects on nurses’ performance and practice. Other methodological strengths include the updated systematic and comprehensive literature search in several databases. In accordance with recommendations [21], we included studies proved too heterogenous for meta-analysis and we chose the vote count method to enhance transparency and clarity of synthesis. However, this method also introduces some limitations. A vote is counted as beneficial based on direction of the effect on study outcomes without considering the statistical significance or magnitude of the results. Three of the included studies had control groups, but the vote count methodology limited the possibility of comparing TTT models to alternative models. Although the synthesis suggested that TTT interventions can increase nurses’ knowledge, we were unable to synthesise whether alternative training models improved knowledge more effectively. Finally, most included studies were conducted in European or North American healthcare settings and the findings may not be generalizable to countries with different healthcare systems or educational traditions.

## Conclusions

Our systematic review synthesis showed that TTT-programs targeting nurses, social and healthcare assistants/nurse aids can effectively disseminate knowledge from trainers to trainees supporting the underlying assumption of the model that local professionals can be trained to train other peers. Given the nurse shortages and high work pressures TTT models may be a timesaving and sustainable way of delivering education. However, the methodological limitations identified in this review (e.g., study design, outcome measurements) point out that there is not yet sufficient evidence to conclude whether TTT-programs are more effective compared to other programs. New studies that compare the effectiveness of TTT-programs on high quality measurements with other programs can clarify whether TTT-programs are more sustainable and cost-effective than other programs (e.g., e-learning). Qualitative studies can further illuminate how TTA programs may change practice outcomes. In light of the limited evidence, our findings can nevertheless give healthcare providers insights into the advantages and disadvantages of implementing TTT models in high strung healthcare systems.

### Supplementary Information

**Additional file 1.** Search documentation.

**Additional file 2.** Studies in other languages than English, Danish, Swedish and Norwegian (Languages understood by the review team).

## Data Availability

Search strings are included in Additional file 1 and other data in the current study are available from the corresponding author on request.

## Abbreviations

CAT: Critical appraisal tools
JBI: Joanna Briggs Institute
PICO: Population, Intervention, Comparison, Outcome
PRISMA: Preferred Reporting Items for Systematic Reviews and Meta-Analyses
PRISMA-S: Statement for Reporting Literature Searches in Systematic Reviews
RCTs: Randomized controlled trials
SWiM: Synthesis Without Meta-analysis
TTT: Train-the-trainer
