# Supplementary material for: The impact of train-the-trainer programs on the continued professional development of nurses: a systematic review
Source: BMC Med Educ. 2024 Jan 4;24:30. doi: 10.1186/s12909-023-04998-4 (PMC10768131; doi:10.1186/s12909-023-04998-4)
Supplement: Supplementary file 1 — Additional file 1. Search documentation. [file 12909_2023_4998_MOESM1_ESM.docx]

Search documentation

Key concepts in the search strategy

Concept 1: Train-the-trainer education

The concept of 'train-the-trainer' was searched for in two parts:

1. Relevant subject headings, e.g. the Medical Subject Heading (MeSH) *Teacher Training*.
2. Free-text searches in the title, abstract and keyword fields. We used established concepts, e.g.' cascade training' and key terms, e.g. 'train' and 'trainer' combined with a proximity operator, e.g. 'adj3'.

Concept 2: Healthcare professionals

The concept of 'healthcare professionals' was searched for in two parts:

1. Relevant subject headings, e.g. the Medical Subject Heading (MeSH) *Health Personnel,* exploded to contain all subject headings below.
2. Free-text searches in the title, abstract and keyword fields. We used key terms, e.g. 'healthcare' and 'professional' combined with a proximity operator, e.g. 'adj3'.

Concepts not used

Diabetes Mellitus

Our initial plan was to search for research articles focused on diabetes mellitus only. This was changed after our preliminary searches because:

1. through the search process, we also searched for train-the-trainer courses in other healthcare fields and found that these articles had similar learning outcomes and were therefore considered relevant.
2. In the initial MEDLINE search, which was limited to diabetes mellitus, there were no relevant records among the first 200.

Information sources and methods

Initially, we have searched the databases MEDLINE, Embase, CINAHL and ERIC from inception to 21 January 2020. An updated search in all four databases was conducted on 10 September 2021.

Contact with authors

If no full-text report was available online, it could not be obtained from the Danish Royal Library, or if relevant data was missing from the full-text report and its supplementary material, we sent an e-mail to the first or corresponding author of the report. The author was then given one month to answer before the report was excluded. All was used if the author had more than one e-mail address listed.

Searches and results

All complete search strategies with results and comments are listed in table 1. This includes the added date limit used for the updated search. Because the Danish Royal Library changed its access to the database ERIC, the initial search was done in the EBSCO interface and the updated search in the ProQuest interface.

Table 1. Searches for all four database searches.

| # | [MEDLINE (Ovid)] | | # | [Embase (Ovid)] | | # | [CINAHL (EBSCO)] | | # | [ERIC (EBSCO)] | | Comments |
| --- | --- | --- | --- | --- | --- | --- | --- | --- | --- | --- | --- | --- |
| *Train-the-trainer* | | | | | | | | | | | | |
| 1 | Teacher Training/ | 228 | 1 | teacher training/ | 374 |  |  |  |  |  |  | Subject headings for the intervention of interest (train-the-trainer education). |
| 2 | ((coaching or coached or educate or educating or educated or guiding or guided or instruct or instructing or instructed or teach or teaching or taught or train or training or trained or tutoring or tutored) adj3 (coach or coaches or educator$ or guide or guides or instructor$ or teacher$ or trainer$ or tutor or tutors)).ti,ab,kf,kw. | 9647 | 2 | ((coaching or coached or educate or educating or educated or guiding or guided or instruct or instructing or instructed or teach or teaching or taught or train or training or trained or tutoring or tutored) adj3 (coach or coaches or educator$ or guide or guides or instructor$ or teacher$ or trainer$ or tutor or tutors)).ti,ab,kw. | 12717 | 1 | TI ((coaching or coached or educate or educating or educated or guiding or guided or instruct or instructing or instructed or teach or teaching or taught or train or training or trained or tutoring or tutored) N3 (coach or coaches or educator* or guide or guides or instructor* or teacher* or trainer* or tutor or tutors)) OR AB ((coaching or coached or educate or educating or educated or guiding or guided or instruct or instructing or instructed or teach or teaching or taught or train or training or trained or tutoring or tutored) N3 (coach or coaches or educator* or guide or guides or instructor* or teacher* or trainer* or tutor or tutors)) | 7,960 | 1 | TI ((coaching or coached or educate or educating or educated or guiding or guided or instruct or instructing or instructed or teach or teaching or taught or train or training or trained or tutoring or tutored) N3 (coach or coaches or educator* or guide or guides or instructor* or teacher* or trainer* or tutor or tutors)) OR AB ((coaching or coached or educate or educating or educated or guiding or guided or instruct or instructing or instructed or teach or teaching or taught or train or training or trained or tutoring or tutored) N3 (coach or coaches or educator* or guide or guides or instructor* or teacher* or trainer* or tutor or tutors)) OR KW ((coaching or coached or educate or educating or educated or guiding or guided or instruct or instructing or instructed or teach or teaching or taught or train or training or trained or tutoring or tutored) N3 (coach or coaches or educator* or guide or guides or instructor* or teacher* or trainer* or tutor or tutors)) | 75,220 | Free-text terms for the intervention of interest (train-the-trainer education). This search line combines the act of teaching with the person being taught with a proximity operator. |
| 3 | ((coach or coaches or guide or guides or tutor or tutors) adj3 (educator$ or instructor$ or teacher$ or trainer$)).ti,ab,kf,kw. | 1204 | 3 | ((coach or coaches or guide or guides or tutor or tutors) adj3 (educator$ or instructor$ or teacher$ or trainer$)).ti,ab,kw. | 1487 | 2 | TI ((coach or coaches or guide or guides or tutor or tutors) N3 (educator* or instructor* or teacher* or trainer*)) OR AB ((coach or coaches or guide or guides or tutor or tutors) N3 (educator* or instructor* or teacher* or trainer*)) | 1,093 | 2 | TI ((coach or coaches or guide or guides or tutor or tutors) N3 (educator* or instructor* or teacher* or trainer*)) OR AB ((coach or coaches or guide or guides or tutor or tutors) N3 (educator* or instructor* or teacher* or trainer*)) OR KW ((coach or coaches or guide or guides or tutor or tutors) N3 (educator* or instructor* or teacher* or trainer*)) | 15,484 | Free-text terms for the intervention of interest (train-the-trainer education). This search line is added because some words could be placed on both sides of the adjacency operator and thereby retrieve multiple references of no relevance. |
| 4 | (coach-the-coach$ or pass-it-on or ttt or t-t-t or tutor-the-tutor$ or train-the-trainer$ or cascade training).ti,ab,kf,kw. | 2586 | 4 | (coach-the-coach$ or pass-it-on or ttt or t-t-t or tutor-the-tutor$ or train-the-trainer$ or cascade training).ti,ab,kw. | 3791 | 3 | TI (coach-the-coach* or pass-it-on or ttt or t-t-t or tutor-the-tutor* or train-the-trainer* or cascade training) OR AB (coach-the-coach* or pass-it-on or ttt or t-t-t or tutor-the-tutor* or train-the-trainer* or cascade training) | 1,170 | 3 | TI (coach-the-coach* or pass-it-on or ttt or t-t-t or tutor-the-tutor* or train-the-trainer* or cascade training) OR AB (coach-the-coach* or pass-it-on or ttt or t-t-t or tutor-the-tutor* or train-the-trainer* or cascade training) OR KW (coach-the-coach* or pass-it-on or ttt or t-t-t or tutor-the-tutor* or train-the-trainer* or cascade training) | 599 | Free-text terms for the intervention of interest (train-the-trainer education). This search line captures fixed concepts and abbreviations. |
| 5 | or/1-4 | 12699 | 5 | or/1-4 | 16787 | 4 | S1 OR S2 OR S3 | 9,532 | 4 | S1 OR S2 OR S3 | 87,973 |  |
| *Health personnel* | | | | | | | | | | | | |
| 6 | exp Health Personnel/ | 500695 | 6 | exp health care personnel/ | 1504714 | 5 | (MH "Health Personnel+") | 507,309 | 5 | DE "Health Personnel" OR DE "Allied Health Personnel" OR DE "Mental Health Workers" OR DE "Nurses" OR DE "Physicians" OR DE "Psychologists" | 15,052 | Subject headings for the population of interest (healthcare professionals). |
| 7 | (((care$ or health$ or medical$ or healthcare$) adj (assistant$ or personnel$ or provider$ or worker$ or giver$ or professional$)) or nurse$ or doctor$ or physician$ or caregiver$).ti,ab,kf,kw. | 870512 | 7 | (((care$ or health$ or medical$ or healthcare$) adj (assistant$ or personnel$ or provider$ or worker$ or giver$ or professional$)) or nurse$ or doctor$ or physician$ or caregiver).ti,ab,kw. | 1098597 | 6 | TI ((care* or health* or medical* or healthcare*) W1 (assistant* or personnel* or provider* or worker* or giver* or professional*) or nurse* or doctor* or physician* or caregiver*) OR AB ((care* or health* or medical* or healthcare*) W1 (assistant* or personnel* or provider* or worker* or giver* or professional*) or nurse* or doctor* or physician* or caregiver*) | 550,525 | 6 | TI (((care* or health* or medical* or healthcare*) W1 (assistant* or personnel* or provider* or worker* or giver* or professional*)) or nurse* or doctor* or physician* or caregiver*) OR AB (((care* or health* or medical* or healthcare*) W1 (assistant* or personnel* or provider* or worker* or giver* or professional*)) or nurse* or doctor* or physician* or caregiver*) OR KW (((care* or health* or medical* or healthcare*) W1 (assistant* or personnel* or provider* or worker* or giver* or professional*)) or nurse* or doctor* or physician* or caregiver*) | 40,323 | Free-text terms for the population of interest (healthcare professionals). |
| 8 | 6 or 7 | 1153195 | 8 | 6 or 7 | 2084017 | 7 | S5 OR S6 | 891,231 | 7 | S5 OR S6 | 48,437 |  |
|  |  |  | 9 | 5 and 8 | 6098 |  |  |  |  |  |  | Combining the two key concepts (train-the-trainer, healthcare professionals) in Embase. |
| *Effectiveness filter* | | | | | | | | | | | | |
|  |  |  | 10 | limit 9 to (randomized controlled trial or controlled clinical trial) | 314 |  |  |  |  |  |  | Limits for the study type of interest (effectiveness studies). |
|  |  |  |  |  |  | 8 | (MH "Randomized Controlled Trials+") OR (MH "Evaluation Research+") | 225,912 |  |  |  | Subject headings terms for the study type of interest (effectiveness studies). |
| 9 | ("randomized controlled trial" or "controlled clinical trial" or "evaluation studies" or "pragmatic clinical trial").pt. | 835200 |  |  |  | 9 | PT (Randomized Controlled Trial) OR PT (Nursing Interventions) | 86,199 |  |  |  | Publication types for the study type of interest (effectiveness studies). |
| 10 | (effect$ or evaluat$ or random$ or trial).ti,ab,kf,kw. | 9415427 | 11 | (effect$ or evaluat$ or random$ or trial).ti,ab,kw. | 11879445 | 10 | TI (effect* or evaluat* or random* or trial) OR AB (effect* or evaluat* or random* or trial) | 77,312 | 8 | TI (effect* or evaluat* or random* or trial) OR AB (effect* or evaluat* or random* or trial) OR KW (effect* or evaluat* or random* or trial) | 519,752 | Free-text terms for the study type of interest (effectiveness study). |
| 11 | 9 or 10 | 9577323 | 12 | 9 and 11 | 3505 | 11 | S8 OR S9 OR S10 | 251,521 |  |  |  |  |
| 12 | 5 and 8 and 11 | 1846 | 13 | 10 or 12 | 3511 | 12 | S4 AND S7 AND S11 | 198 | 9 | S4 AND S7 AND S8 | 839 | Combining the two key concepts (train-the-trainer, healthcare professionals) and the effectiveness filter. |
| *Animal filter* | | | | | | | | | | | | |
| 13 | exp Animals/ not Humans/ | 4666040 | 14 | (exp animal/ or nonhuman/) not exp human/ | 6278923 |  |  |  |  |  |  | Excluding animal studies from the search result. |
| 14 | 12 not 13 | 2006 | 15 | 13 not 14 | 3502 |  |  |  |  |  |  |  |
| *Limit to exclude medline journals* | | | | | | | | | | | | |
|  |  |  | 16 | limit 15 to exclude medline journals | 289 |  |  |  |  |  |  | Excluding MEDLINE journals from the search result in Embase. |
| *Updated search* | | | | | | | | | | | | |
| 15 | limit 14 to (dt="20200120-20251231" or ez="20200120-20251231") | 302 | 17 | limit 16 to (dc="20200120-20251231" or rd="20200120-20251231") | 119 | 13 | EM 20200120-20251231 | 21 | 10 | yr(2020-2029)  (Updated search made in ProQuest) | 26 | Updating search using date filters. |
