# Supplementary material for: The impact of train-the-trainer programs on the continued professional development of nurses: a systematic review
Source: BMC Med Educ. 2024 Jan 4;24:30. doi: 10.1186/s12909-023-04998-4 (PMC10768131; doi:10.1186/s12909-023-04998-4)
Supplement: Supplementary file 2 — Additional file 2. Studies in other languages than English, Danish, Swedish and Norwegian (Languages understood by the review team). [file 12909_2023_4998_MOESM2_ESM.docx]

Studies in other languages than English, Danish, Swedish and Norwegian (languages understood by the review team).

Christ G, Dressel R and Reiter-Theil S ;. (1995). ["Teachers' Training Course"--evaluation of continuing studies for faculty in the field of ethics in medicine].. *"Teachers' Training Course"--Evaluation eines Fortbildungsangebots fur Lehrende auf dem Gebiet der Ethik in der Medizin.*, 89(4), pp.337-9.

Giusti Angela, Conti Stefania and Di Lorenzo ; Giuseppina ; Donati Serena ; Perra Alberto ; Grandolfo Michele ;. (2006). [How Italian midwives contribute to breastfeeding promotion: a national experience of "cascade" training].. *L'apporto delle ostetriche italiane nella promozione dell'allattamento al seno: un'esperienza nazionale di formazione a cascata.*, 62(1), pp.53-67.

Homeier I and Lichtenschopf A . (1999). The Train-the-Trainer-model for health-care assistants from the working group for patient education of the OGLUT. *Das Train-the-Trainer-modell fur ordinationsassistentinnen der osterreichischen arbeitsgruppe fur patientenschulung der OGLUT*, 25(12), pp.698-703.

Konig Sarah, Stieger Philipp and Sippel Sonia ; Kadmon Martina ; Werwick Katrin ; Sterz Jasmina ; Hoefer Sebastian H; Russeler Miriam ; Walcher Felix ; Adili Farzin ;. (2019). [Train-the-Trainer: Professionalisation of Didactics in Daily Clinical Routine - the Personal Perception of Clinical Teaching Staff with Respect to Didactic Competence and the Framework Conditions of Teaching].. *Train-the-Trainer: Professionalisierung der Lehre im klinischen Alltag - Selbsteinschatzung Lehrender zur didaktischen Kompetenz und den Rahmenbedingungen des Unterrichts.*, 144(6), pp.551-559.
